# Development and validation of a gene expression oligo microarray for the gilthead sea bream (*Sparus aurata*)

**DOI:** 10.1186/1471-2164-9-580

**Published:** 2008-12-03

**Authors:** Serena Ferraresso, Nicola Vitulo, Alba N Mininni, Chiara Romualdi, Barbara Cardazzo, Enrico Negrisolo, Richard Reinhardt, Adelino VM Canario, Tomaso Patarnello, Luca Bargelloni

**Affiliations:** 1 Department of Public Health, Comparative Pathology and Veterinary Hygiene, Faculty of Veterinary Medicine, University of Padova, Viale dell'Università 16, 35020 Legnaro, Italy; 2 CRIBI, University of Padova, Complesso Biologico Vallisneri, Via Ugo Bassi 58/B, Padova, Italy; 3 Max Planck Institute for Molecular Genetics, Ihnestraße 63-73, 14195 Berlin, Germany; 4 Centro de Ciências do Mar, Universidade do Algarve, Gambelas, 8005-139 Faro, Portugal

## Abstract

**Background:**

Aquaculture represents the most sustainable alternative of seafood supply to substitute for the declining marine fisheries, but severe production bottlenecks remain to be solved. The application of genomic technologies offers much promise to rapidly increase our knowledge on biological processes in farmed species and overcome such bottlenecks. Here we present an integrated platform for mRNA expression profiling in the gilthead sea bream (*Sparus aurata*), a marine teleost of great importance for aquaculture.

**Results:**

A public data base was constructed, consisting of 19,734 unique clusters (3,563 contigs and 16,171 singletons). Functional annotation was obtained for 8,021 clusters. Over 4,000 sequences were also associated with a GO entry. Two 60mer probes were designed for each gene and *in-situ *synthesized on glass slides using Agilent SurePrint™ technology. Platform reproducibility and accuracy were assessed on two early stages of sea bream development (one-day and four days old larvae). Correlation between technical replicates was always > 0.99, with strong positive correlation between paired probes. A two class SAM test identified 1,050 differentially expressed genes between the two developmental stages. Functional analysis suggested that down-regulated transcripts (407) in older larvae are mostly essential/housekeeping genes, whereas tissue-specific genes are up-regulated in parallel with the formation of key organs (eye, digestive system). Cross-validation of microarray data was carried out using quantitative qRT-PCR on 11 target genes, selected to reflect the whole range of fold-change and both up-regulated and down-regulated genes. A statistically significant positive correlation was obtained comparing expression levels for each target gene across all biological replicates. Good concordance between qRT-PCR and microarray data was observed between 2- and 7-fold change, while fold-change compression in the microarray was present for differences greater than 10-fold in the qRT-PCR.

**Conclusion:**

A highly reliable oligo-microarray platform was developed and validated for the gilthead sea bream despite the presently limited knowledge of the species transcriptome. Because of the flexible design this array will be able to accommodate additional probes as soon as novel unique transcripts are available.

## Background

The gilthead sea bream (*Sparus aurata *Linnaeus, 1758) is a marine teleost that belongs to the family Sparidae. Sparids are of great importance for fisheries and aquaculture, being excellent food fish, with high commercial value *S. aurata *is one of the most prominent, with an average cultured production of 100 million metric tonnes per year. The great importance of the gilthead sea bream for marine aquaculture has fuelled an increasing number of studies in many different areas such as immunology, endocrinology, bone morphology, and muscle physiology. Furthermore, the genomic toolkit for this species has been constantly improving in the recent years. A first generation cDNA microarray was recently reported [1], a radiation hybrid (RH) map has been constructed [2] and further improved with over 1,000 markers [3]. A medium density genetic linkage map is already available [4], a second generation linkage map is being constructed (L. Bargelloni unpublished data), and a BAC-end sequencing project is underway (R. Reinhardt unpublished data).

Despite great achievements in marine fish culture, severe bottlenecks still remain (*e.g*. high larval mortality, skeletal malformations, susceptibility to stress and disease). To overcome these limitations, important gaps need to be filled in the basic knowledge of biology for aquacultured species. A better understanding of the molecular mechanisms underlying key productive traits (*e.g*. growth rate, muscle and bone development, resistance/susceptibility to stress and disease) holds the promise to revolutionize animal farming, leading to improved programs of genetic breeding and highly effective means to monitor the effects of husbandry conditions on farmed animals. Functional genomics, *i.e. *a "whole-genome" approach to the study of interactions between genes and environment, offers unprecedented opportunities to achieve such a goal. Not surprisingly, relevant "genomic" research programs have been launched for all the most important livestock species. Large collections of ESTs have been produced (*e.g*. 1,560,130 ESTs for cattle, 2,227,253 for pig, 632,013 for chicken [5]), and technical platforms for functional genomics, based on DNA microarrays are now available (*e.g. *Affymetrix or Agilent oligo-DNA microarrays). With respect to farmed fish, only recently large sequencing efforts led the improvement of EST collections for several species such as Atlantic salmon [6], rainbow trout [7], Atlantic cod, Atlantic halibut [8], channel and blue catfish [9], largemouth bass [10], and fathead minnow [11]. Such large collections of sequence data cannot be fully exploited to develop functional genomic tools using the traditional cDNA microarray technology.

Even neglecting other technical limitations, cDNA arrays require to be produced that all the clones to be spotted onto the slide are physically available at a single location. This often led to the construction of cDNA microarrays that provided only a partial representation of the species transcriptome, focused to restricted research goals [12-14]. Furthermore, ultra-high throughput DNA sequencing technologies (e.g. 454), which can now produce up to one million ESTs in a single run [15,16], do not use individual bacterial clones as sequencing material. Therefore, amplifying and spotting cDNA clones is not possible anymore. Oligo_DNA arrays have long offered an alternative approach to cDNA arrays, allowing the representation of all the expressed sequences that are available for the target species. Oligonucleotide probes of variable length (24–70mer) can be either synthesized individually and then spotted onto the slide or directly synthesized *in situ*. Until recently, a large economic investment was associated with the development of oligo-DNA arrays as a consequence of the cost of individual oligo synthesis or the development of a specific photolithographic mask (Affymetrix). The advent of different technologies (*e.g. *Nimblegen, Agilent, Combimatrix) that allow flexible in situ probe synthesis has made affordable the development of high-density oligo-DNA microarrays also in non-model species. In fact, this year two generations of oligo-DNA microarrays, based on short (24mer) probes have been developed for ictalurid catfish using Nimblegen technology [17-19]; Parallel Synthesis Technology has been used to fabricate a high-density DNA microarray for Atlantic halibut [20], while Agilent SurePrint™ Technology has been applied to produce platforms, different both in size and probe-length, for the fathead minnow [21-23], the largemouth bass [10], and the rainbow trout [24,25]. A low-density oligo-DNA microarray (5k) has been tested also for the Atlantic salmon [26]. In the present study, we used all the available ESTs from the gilthead sea bream to design two longer (60mer) probes for each transcript *in-situ *synthesized on glass slides using Agilent SurePrint™ technology. This microarray platform was then validated to assess its reproducibility and accuracy on two early stages of gilthead sea bream development, respectively one-day and four days old larvae.

## Results

### SAPD data base

**Figure 1:**
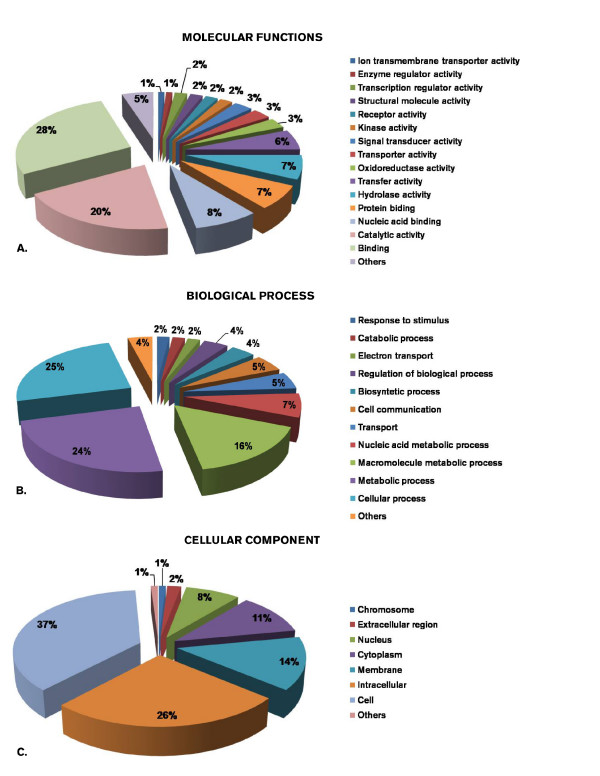
**Percentage distribution of the GO entries associated to sea bream transcripts**. Most represented entries within A: MOLECULAR FUNCTION; B: BIOLOGICAL PROCESS; C: CELLULAR COMPONENT.

A total of 59,485 ESTs plus 157 sea bream mRNA sequences publicly available in GenBank were clustered together. The number of unique clusters was 19,734. Contigs formed by two or more ESTs were 3,563, while singletons were 16,171. The relevant number of singletons is likely due to two factors. First, highly stringent criteria were enforced during the assembly process (see Methods) in order to avoid the assembly of concatamers. Second, only normalized cDNA libraries were used to produce the vast majority of clustered ESTs, this determined a few number of contigs compared to singletons. The annotation process identified 8,021 clusters with a significant similarity to a known gene. To a large proportion of contigs it was also possible to associate a GO entry, either for Biological process (3,332 clusters) or Cellular component (2,301 clusters) or Molecular function (4,420 clusters) (see Figure 1). A total of 7,913 clusters encode a putative protein with one or more known Pfam domains. All sequence data with associated annotations are stored in a dedicated data base (SAPD database: [27]), which is freely accessible. The data base is based on the BioMart environment, which allows several options for data mining and retrieval. A local Blast search is also implemented.

### Microarray analysis

Probe design was positively completed for 19,715 target clusters. Of these, 19,664 were represented by two non-overlapping probes, for 51 it was possible to design only one probe. A total of 39,379 target probes were then synthesized directly onto the glass slide. The majority of designed probes (96.1%) had the highest quality score (BC1), 3.6% were scored as BC2, the remaining ones (0.3%) had BC3 or BC4 scores, none showed the lowest score (BC-poor).

The quality of each probe included on the array was then assessed for hybridization success considering a total of 10 experiments (5 biological replicates for each of the two *S. aurata *developmental stages tested). Hybridizations resulting in a "present" flag using the Agilent *Feature Extraction 9.5.1 *software were considered successful. Only five probes (0.013%) never showed higher signal than background, while 37,585, corresponding to 95% of the total number of target probes, successfully hybridized in at least five array experiments [see Additional file 1].

One of the most important requirements for a microarray experiment is good system reproducibility, which ensures that results from different experiments can be directly and reliably compared. Four technical replicates of the same experiment were performed in order to evaluate the repeatability and precision of the experimental protocol and of the array platform. Raw expression data were filtered according to missing spot intensities *per *probe. Probes with more than one missing spot across the four replicates were removed from the analysis (3,224 probes removed, 8% of the total number of probes). After data filtering and cyclic lowess normalization, the degree of mutual agreement among replicates was estimated using Pearson correlation coefficients on the entire set of expression values. For all pairs of experiments correlation coefficients were always significant (*p-value *< 1E-5) and never less than 0.99 (almost a perfect correlation). This underlines the high level of repeatability for this array platform. The % coefficients of variation (CV) of the normalized signals at the feature level were measured across non-control probes of the four replicated microarray. Median %CV was 1.1%, and less than 15% of the probes had a CV over 30% (2,562 on 37,592).

**Figure 2:**
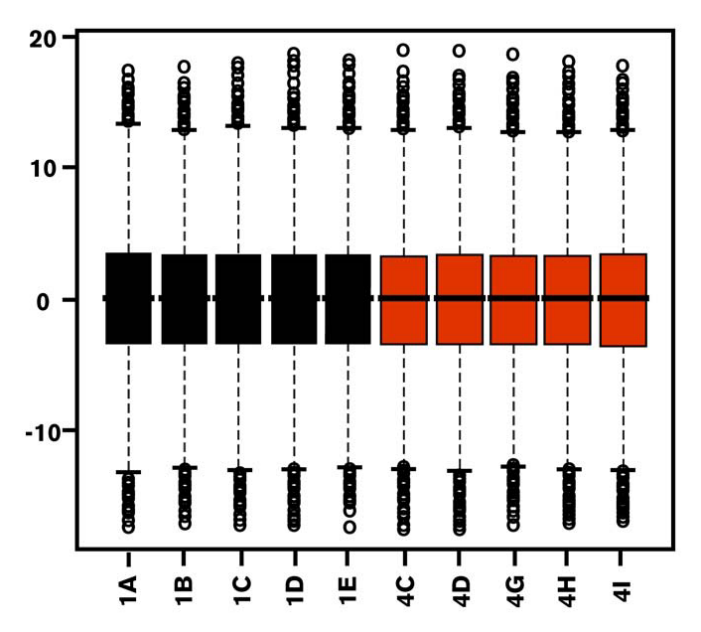
**Boxplots of observed fold-changes for comparison between Probe_1 and Probe_2 across all the experiments**. The boxplots show the distribution of observed Fold-change (y axis) between the two probes for each transcript. Labels on the x-axis refer to the following experiments: 1A-1E, biological replicates of Stage 1; 4C-4I, biological replicates of Stage 4.

**Figure 3:**
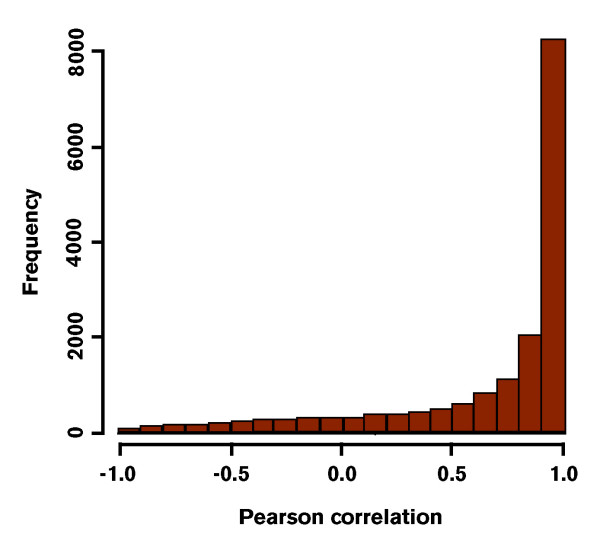
**Correlation between levels of gene expression measured by Probe_1 and Probe_2**. For each gene, the Pearson correlation coefficient was calculated within and among arrays.

As for technical replicates, raw expression data derived from the comparison of the two *S. aurata *developmental stages, were filtered according to missing spot intensity. Probes with more than two missing values across the biological replicates of each developmental stage were removed from the analysis (4,846 probes removed, 12% of the total number of target probes). Then, cyclic lowess was used to normalize the data. The gilthead sea bream microarray platform is characterised by the presence of two probes for each transcript. These two probes match the gene sequence at two non-overlapping positions. In particular, the first probe was designed to be closer to the 3' of each target gene. The variability between the two probes for the same transcript was assessed using fold change as measure of signal difference. Such comparisons are expected to yield a fold change close or equal to zero. In Figure 2 each plot shows the distribution of observed fold-changes between Probe_1 and Probe_2 for individual array experiments. As expected, the difference between the intensities of the two probes for the same gene displays a symmetrical distribution centred on zero and equal across all the experiments. With the exception of a few cases, most probe pairs are characterised by a small difference in terms of intensity values. To evaluate the degree of concordance for expression values of probe pairs, a correlation analysis was carried out. For each gene, the Pearson correlation coefficient was calculated within and among arrays. Within arrays the total expression values of Probe_1 and Probe_2 showed a correlation coefficient always greater than 0.8. On the other hand, the correlation among arrays was evaluated using, respectively, vectors of Probe_1 and Probe_2 expression values across all ten experiments. The distribution of correlation coefficients (see Figure 3) indicates that most probes (68%) have a strong positive correlation (*r *> 0.7), 11% show a moderate correlation (0.5 <*r *< 0.7), while only a small proportion of probes are negatively correlated (3%), some of them (1%) with a strong negative correlation, *r *< -0.7).

A microarray platform should also cover a wide dynamic range to detect/quantify both rare and abundant genes in the same experiment. Sensitivity and dynamic range of the platform were measured using the *Spike-in *control probes. *Spike-in *mix contains a mixture of 10 *in vitro *synthesized, poly-adenilated transcripts, derived from Adenovirus E1A gene, at concentrations that span six logs (from 0.04 pg/μl to 40,000 pg/μl). When the signal intensity (processed signal) for each *Spike-in *transcript is plotted against the log of the relative concentration, the linear range can be calculated based on parametric curve-fit through the data. The lower limit of detection (LLD) of the microarray experiments was estimated using the lowest intensity probe within the linear range. In all experiments a large dynamic range was observed with linear increase in signal intensity across 5 (4.96 ± 0.2) orders of magnitude, and a lower LLD of 0.4 pg/μl (corresponding to *Spike-in *probe E1A_r60_a104). The transcript with the lowest concentration (E1A_r60_3, corresponding to 0.04 pg/μl) was always out of the linear range due to its extremely low signal intensity.

A two class SAM test [28] was performed to identify differentially expressed genes between developmental stages 1 and 4, with a False Discovery Rate (FDR) equal to zero. This produced a list of 1,518 (4%) significant probes corresponding to 1,050 unique genes. For 468 out of 1,050 genes both Probe_1 and Probe_2 resulted differentially expressed after SAM analysis while the remaining 582 genes were represented by only one probe. For 41 genes (out of 582) identified by a single probe the other one was previously excluded in the filtering step. Transcripts that were up-regulated in Stage 4 compared to Stage 1 were 643 (289 with both probes), while down-regulated genes were 407 (179 with two probes). A preliminary annotation was available respectively for 133 (21%) up-regulated genes, whereas a significantly larger proportion (Fisher-exact test p < 0.0001) of down-regulated genes was associated with an annotation (283, 70%) [see Additional file 2]. A GO definition of the biological process associated with the encoded protein was obtained for 134 (33%) of down-regulated transcripts. Of these, 38 are involved in DNA replication or repair, chromatin assembly, and cell cycle regulation, while 36 are part of protein synthesis/maturation (18) or protein catabolism (18) processes. The third most represented group is lipid transport and metabolism (12). Conversely, only 52 (8%) up-regulated genes are associated with a GO definition of biological process. The most represented group (proteolysis, 9 entries) contains proteases with various functions, *e.g. *digestive enzymes (chymotrypsinogen, elastase) or antigen processing peptidases (cathepsin L1). Signal transduction is the second most frequent process, with 7 entries. Noteworthy are two proteins involved in phototransduction (retinal cone arrestin-3 and green-sensitive opsin-1) and the nuclear receptor for glucocorticoids (Nuclear receptor 3 C1). Other GO biological process categories with fewer entries are "metabolic process" with 6 entries, mostly consisting of carbohydrate processing enzymes, and "transport" (6 transcripts) with transporters/channels for diverse molecules (ions, lipids). A single entry (MHC class IIA antigen) was present for "immune response", a crucial biological process for larval survival.

Raw and normalized fluorescence data have been deposited in the GEO data base under accession numbers GSM305530, GSM305531, GSM305544, GSM305551 (series GSE12116 and GSE12118).

### Real-time RT-PCR analysis

**Figure 4:**
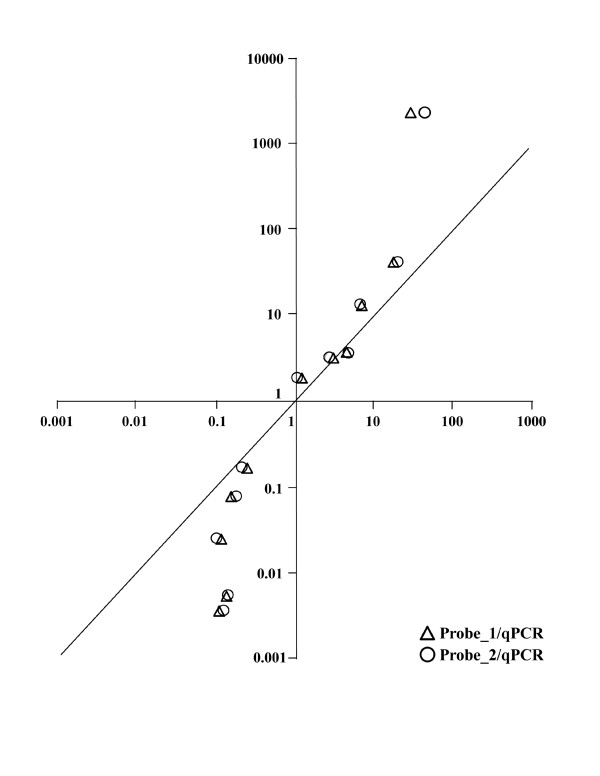
**Comparison between microarray and qPCR results**. Expression values for the eleven target genes were compared between microarray probes and Real-time RT-PCR data. Triangles: ratio between Probe_1- and qPCR-estimated fold-changes. Circles: ratio between Probe_2- and qPCR-estimated fold-changes.

To cross-validate platform performance, gene-specific quantitative qRT-PCR assays, designed using the Universal Probe Library (UPL) system, were used. Target genes for qRT-PCR analysis were selected according to the following selection criteria. Selected genes (i) should reflect the whole range of fold-change values (1.25–44) (ii) should equally represent up-regulated and down-regulated gene lists, (iii) should be present with both independent probes in the normalized data set. Table 1 shows fold-changes detected by gene-specific PCR assay and by both microarray probes (1 and 2) for the same target transcript. Fold-change was calculated as the ratio of mean signal intensity across five biological replicates between Stage 4 and Stage 1. For all tested targets, the direction of change in expression was concordant between qRT-PCR and microarray results. Good concordance between qRT-PCR and microarray data was observed when fold-change values ranged between 2 and 7. When the fold-change calculated from microarray data was higher than 10, qRT-PCR estimated substantially larger changes in gene-expression (Figure 4). Overall, a statistically significant correlation was obtained comparing expression levels for each target gene across all biological replicates. Six genes showed high correlation coefficients (Spearman rho > 0.8) for both probes (p < 0.01) with qPCR data (Table 2). Other four genes had a significant correlation (0.6 < rho < 0.8 with p < 0.05). Only one gene, PGK1, presented a not significant, albeit positive correlation (rho = 0.5, p > 0.1) for one probe, and no correlation for the other one (rho = -0.04).

**Table 1:** Comparison of fold-change values from qRT-PCR and microarray for selected target genes.

**TARGET TRANSCRIPT**	**SAPD ID**	**Fold Change**^a^		
		**Real-time RT-PCR**	**Microarray Probe_1**	**Microarray Probe_2**
Apolipoprotein E1	SAPD02358	0.00362	0.12263	0.1276
Flap endonuclease 1	SAPD04884	0.07811	0.16678	0.18717
Ovostatin	SAPD01680	0.00525	0.13731	0.14149
Myosin	SAPD10294	0.16533	0.25064	0.21589
Serotransferrin	SAPD01126	0.02439	0.11883	0.10408
Glutamate R7	SAPD19202	3.58394	4.30343	4.61982
Methionine aminopeptidase 2	SAPD26496	12.9384	6.88419	6.91868
L-lactate dehydrogenase	SAPD00597	3.09142	2.99927	3.00815
Serine racemase	SAPD19150	40.9397	18.3846	19.9929
Retinal cone arrestin-3	SAPD02277	2232.6	29.4717	44.2798
Phosphoglycerate kinase 1	SAPD03464	1.84119	1.26289	1.12021
				
Malate dehydrogenase 1	SAPD02236		1.05	0.99

^a ^Fold change is calculated as ratio of Stage 4 vs Stage 1, using mean signal intensity across five biological replicates of each stage. Values below 1.0 indicate down-regulation in Stage 4.

**Table 2:** Correlation between microarray and real-time RT-PCR expression data.

**Gene**	**Spearman's rho qPCR/Probe_1**	**Spearman's rho qPCR/Probe_2**	**Spearman's rho Probe1/Probe_2**
Apolipoprotein E1	0.915**	0.915**	0.976**
L-lactate dehydrogenase	0.697*	0.636*	0.927**
Methionine aminopeptidase 2	0.939**	0.709*	0.806**
Myosin	0.644*	0.767**	0.867**
Retinal cone arrestin-3	0.905**	0.851**	0.952**
Ovostatin	0.855**	0.855**	0.855**
Phosphoglycerate kinase 1	0.515	-0.042	0.685*
Serine racemase	0.950**	0.917**	0.988**
Serotransferrin	0.732*	0.794**	0.891**
Flap endonuclease 1	0.723*	0.608	0.903**
Glutamate R7	0.818**	0.915**	0.915**

* p < 0.05 ** p < 0.01

## Discussion

The aim of the present work was to develop an integrated platform for mRNA expression profiling in the gilthead sea bream. The first step was the construction of a data base of unique transcripts clustering all publicly available mRNA sequences and >50,000 expressed sequence tags (ESTs) originating from a medium-scale EST sequencing project, which had been recently completed, within the framework of the Network of Excellence Marine Genomics Europe. The number of unique clusters obtained is similar to what reported for comparable EST collections in other fish species/stages (stickleback, Japanese medaka, channel catfish, Atlantic halibut, Atlantic salmon, Atlantic cod, fathead minnow [29], and largemouth bass [10]). Approximately 40% of these unique transcripts found a significant similarity with at least one annotated gene/protein present in public data bases (see Methods), in agreement with the percentage of annotated clusters for the largemouth bass (46%, [10]), and slightly lower than the value observed for the pre-smolt Atlantic salmon (50.3%, [6]), the Atlantic halibut (60%, [8]), and the channel catfish (51% [19]). On the other hand, a sufficiently high number of sea bream transcripts could be associated with a GO entry, potentially allowing for the functional analysis of differentially expressed genes. The relatively low number of annotated expressed sequences appears to be a major limitation of most EST sequencing projects in commercial fish, even in those species where the transcriptome has been characterized in greater depth. However, the percentage of annotated transcripts is expected to increase substantially in the near future, when additional draft sequences of fish genomes (*e.g*. Nile tilapia, Atlantic salmon) will become available. Further sequence information for comparative analysis will also arise from the application of ultra-high throughput DNA sequencing technologies to EST production in non-model species.

The relatively small number of ESTs available for *S. aurata *did not seems to affect significantly the efficiency of probe design, as for most clusters two non-overlapping probes could be successfully designed. Moreover, for most target sequences a strong correlation was reported between probe-pairs. Only for 385 transcripts (3%) Probe_1 and Probe_2 showed a negative correlation. Several different factors can account for such observation. First, alternative splicing could produce differentially expressed transcripts for the same gene; such a difference can then be revealed by the use of two independent probes per gene. Second, a greater stability of the 3'-end of some transcript might reduce the signal for the 5'-end probe. However, this seems not to be a general phenomenon because no significant bias was observed between 3'-end probes and 5'-end ones. Finally, high sequence similarity across different genes (*e.g. *recently duplicated loci) might lead to the widely documented problem of probe cross-hybridization or to spurious EST clusters in consequence of assembly errors.

Before normalization and statistical analysis, data for 12% of the total number of probes were removed, following a very stringent criterion (a maximum of two missing spots was allowed for each probe across five biological replicates). Such filtering step was performed to maximize the probability of detecting real differences in gene expression at the expense of some loss of information. Detailed analysis of filtered-out probes shows that 60% of excluded probes in Stage 1 were detected in Stage 4, and *vice versa *65% of missing spots in Stage 4 were present in Stage 1. This observation suggests that differential expression between ontogenetic phases rather than poor probe quality might explain why a relatively large number of probes were excluded. It should also be noted that experimental samples represented two early larval stages, where a certain number of "adult-only" genes might not be expressed at all. Finally, both probes (1 and 2) were excluded from the analysis only for less than 4% of all genes (769). For the majority of transcripts either one (3,308 genes) or two probes (15,638) yielded a positive signal in all experiments. This clearly suggests that a "safe" approach in microarray design should incorporate at least two probes per gene.

Repeatability of microarray data, across either technical or biological replicates, appeared to be quite high and not influenced by the presently limited knowledge of the sea bream transcriptome. Good repeatability for the Agilent and other oligo-array platforms was already reported in a large initiative on microarray quality [30]. The results obtained here further confirm this evidence. In the MAQC evaluation single- and two-colour designs were compared [31]. This comparison indicated that data quality is essentially equivalent between the one- and two-color approaches and strongly suggested that this variable need not be a primary factor in decisions regarding experimental microarray design. Repeatability was extremely good also in the case of the gilthead sea bream array (correlation coefficient > 0.99 across technical replicates). The use of just one dye (Cy3) allows for a simplified experimental design and easier comparison across different experiments. At the same time, labeling with only Cy3 is less expensive and it reduces the risk of ozone-mediated dye degradation, as Cy5 is more sensitive to this ubiquitous contaminant. A single color scheme, however, requires a highly efficient signal normalization across experiments. Based on the comparison of *Spike-in *probe signal between arrays after normalization, cyclic lowess was found to be superior to quantile normalization, and to outperform averaging with median fluorescence value, which is the method suggested by Agilent for one-color array experiments (data not shown). This result is in agreement with evidence reported for other array platforms [32]. In the Agilent array technology, the simplicity and economy of a single color design is coupled with the flexibility of programmable *in-situ *synthesis of oligonucleotide probes. This feature is extremely important especially for non-model species, where the knowledge of the transcriptome is often substantially incomplete. A flexible array design can accommodate the elimination of unsuitable probes and, more importantly, the subsequent inclusion of additional probes as soon as novel unique transcripts are identified.

The quality of the gilthead sea bream oligo-microarray data was also confirmed after qRT-PCR validation of expression results for selected target genes. The use of qRT-PCR for cross-validation of microarray data is generally limited to the most significant differentially expressed genes. In the present study, genes were selected for validation across the entire range of absolute signal intensity and fold-change. Although this approach cannot substitute for systematic qPCR analysis of all target genes as reported in other studies [33], it should provide a less biased comparison between microarray- and RT-PCR-technology. In the case of the gilthead sea bream oligo-array, a highly significant positive correlation was observed when comparing individual expression values, further confirming the reliability of the gilthead sea bream array platform. PGK1 was the only exception. For this gene, a positive, but not significant correlation was observed only between results of Probe_1 and qRT-PCR data. This is likely due to the small difference in expression between the two sample groups (mean fold-change estimated from array data is 1.1–1.2). Lack of correlation between microarray and qRT-PCR for genes exhibiting low levels of change (<1.4 fold) has been commonly reported. Indeed usually a two-fold change is considered as the cut-off below which microarray and qRT-PCR data begin to loose correlation [34]. Plotting microarray-estimated fold-changes against qRT-PCR results (see Figure 4) also showed the occurrence of fold-change compression for differences in expression value above one order of magnitude. This is, however, a well-known phenomenon, due to various technical limitations, including limited dynamic range, signal saturations, and cross-hybridizations of microarrays [33].

As mentioned above, the main focus of the present study was the construction and validation of a microarray platform for the gilthead sea bream. Nevertheless, significant results on the biological process of gilthead sea bream early development were obtained. It should be remarked here that the expression levels of target genes obtained in the present work reflect a mixture of cell types and tissues, as whole larvae were analyzed. Thus, the variation in expression observed in the comparison between Stage 1 and 4 might represent changes in the proportion of different tissues during development rather than changes in specific levels of transcription of target genes. Furthermore, absence of variation in expression may represent the cancelling out of variations in different tissues of opposite signal. Indeed, genes down-regulated in the transition between 1-day old and 4-days old larvae mainly belong to "essential" (housekeeping) biological processes such as DNA replication, cell cycle, and protein synthesis or catabolism. It is therefore likely that as tissue- and cell-differentiation proceeds cell-type and tissue-type specific transcripts start to be produced, leading to a "dilution" of mRNAs encoding housekeeping proteins. A similar effect might cause the observed down-regulation of proteins involved in lipid metabolism, which is essential for cellular and sub-cellular membrane biosynthesis. On the other hand, in Stage 4 larvae the yolk sac is reduced to one-eighth of its original size, with a corresponding reduction in the contribution of yolk lipids as nutrients. Thus, the reduced abundance of mRNAs encoding proteins associated with lipid metabolic processes could actually reflect a transition toward autonomous feeding. In 4-days old larvae mouth opening is initiated, the digestive system is formed, with a lengthened intestine and a pancreatic gland anlage. In keeping with this evidence, digestive enzymes such as elastase, as already reported by Sarropoulou and colleagues [1], and two different isoforms of chymotrypsinogen [see Additional file 2] begin to appear in the list of up-regulated transcripts. Four-days old larvae also start to show a pigmented eye, as mirrored by the expression of green-sensitive opsin and other eye-specific genes (retinal cone arrestin-3, which is supposed to bind photo-activated opsins, or cathepsin L2, involved in corneal development).

Myogenesis is well underway in early larval stages. The differentiation of embryonic and larval muscle fibres involves a complex temporal sequence of gene activation [35-37] that includes structural and contractile proteins (*e.g. *myosin, tropomyosin) as well as soluble muscle proteins and enzymes (*e.g. *parvalbumin, muscle creatine kinase). Unfortunately, little is known on the temporal and spatial organization of gene expression for the maturation and diversification of fish embryonic muscle cells.

In the present study high expression levels of the myogenic regulatory factor MyoD have been detected in both Stage 1 and Stage 4 larvae. Similarly, transcritps encoding proteins involved in muscle contraction such as myosin light chain 1, parvalbumin, tropomyosin, and sarcomeric creatine kinase (ckm) are abundantly expressed. The latter shows strong up-regulation in Stage 4, thus confirming previous findings on the constant increase of ckm expression from the embryo to the adult [35]. Differences of gene expression have been detected also for tropomyosin, increasing in expression as the embryos get older, while myosin and parvalbumin show a weak up-regulation (< 4-fold) in Stage 1 compared to Stage 4, when the larvae has just hatched, as already reported by Sarropoulou and colleagues [1]. Finally, stromal cell derived-factor, a molecule promoting early myogenic differentiation of external cell precursors [38], appears to be down-regulated in Stage 4 compared to Stage 1.

More in general, signal transduction is a well represented biological process among up-regulated genes, indicating an increasing importance of intra-cellular signaling pathways in parallel with tissue- and cell-differentiation. In some cases, the appearance of specific pathways seems to precede that of the corresponding anatomical organs. For instance, the glucocorticoid receptor is up-regulated in agreement with a functional hypothalamus-pituitary-interrenal axis at an early stage [39] and suggesting a role of glucocorticoids in early development. The shift from "essential" transcripts toward tissue- and cell-specific ones might also explain the highly significant bias in the percentage of annotated/unknown transcripts between up-regulated and down-regulated genes. A low frequency (21%) of annotated clusters among up-regulated transcripts in Stage 4 larvae was observed when compared to down-regulated ones (80%). Cluster annotation was based essentially on sequence similarity, therefore sea bream transcripts from highly conserved genes are more likely to find a significant match with known sequences from other taxa. A correlation between sequence conservation and protein function/tissue-distribution/expression has been the focus of several studies [40-44]. It seems, at least in mammals, that essential genes (defined on the basis of gene-ablation studies in mice) or housekeeping genes (ubiquitously expressed genes) evolve significantly slower than non-essential or tissue-specific genes. These two categories do not necessarily coincide, but there is a substantial overlapping. In the case of gilthead sea bream expression data, the transition between Stage 1 and Stage 4 larvae represents an increase in tissue- and cell-types with a correspondingly larger proportion of tissue- and cell-specific transcripts. This likely translates into a higher share of essential/housekeeping genes in Stage 1 than in Stage 4, as already evident from GO biological process entries associated with up-regulated and down-regulated genes. Since a significantly higher number of down-regulated transcripts shows a meaningful similarity with putative homologs in other species, it seems likely that essential/housekeeping genes evolve more slowly in the gilthead sea bream as well. Thus, similar selective processes appear to shape the evolution of protein-encoding genes in both lower and higher vertebrates.

## Conclusion

A highly reliable oligo-microarray platform could be developed and validated for the sea bream despite the presently limited knowledge of the species transcriptome. Strong reproducibility was achieved, and microarray data could be cross-validated using an independent method (qRT-PCR). While usable as it is, because of its flexible design this type of array will be able in the future to accommodate additional probes as soon as novel unique transcripts are identified. Finally, the approach followed here can be extended to any species of interest, especially in conjunction with EST production based on next-generation sequencing. Together with similar studies carried out in other fish, the present work demonstrates that the development of flexible and reliable array platforms is feasible in any important aquaculture species with a limited investment. The possibility to analyze global gene expression profiles under different environmental conditions will lead to a better understanding of the influence of nutrition, stress, and disease on aquaculture production.

## Methods

### Sample collection and RNA extraction

Early developmental stages of gilthead sea bream were collected at the fish farm "Impianto di Acquacoltura Ca' Zuliani" (Monfalcone, Italy), anesthetized, snap frozen in liquid nitrogen, and stored at -80°C. For two stages, Stage 1 (larvae at 24 hours post-hatching) and Stage 4 (larvae at 96 hours post-hatching) total RNA was extracted from five independent pools per stage using the RNAeasy Mini Kit (Qiagen, Hilden, Germany). Each pool contained approximately 40–50 larvae. An additional pool was prepared mixing larvae of four different stages, the RNA extracted as described above, and used to prepare four technical replicates to test array-to-array reproducibility of the hybridization step. RNA quality was preliminarily checked by gel electrophoresis on a 1% agarose gel containing SYBR Safe™ DNA Gel stain 10,000× (Invitrogen™, Carlsbad, California).

RNA concentration was also determined using a UV-Vis spectrophotometer NanoDrop^® ^ND-1000 (NanoDrop Technologies, Wilmington, USA). RNA integrity and quality was then estimated on Agilent 2100 Bioanalyzer (Agilent Technologies, Palo Alto, CA) and RNA integrity number (RIN) index was calculated for each sample using the Agilent 2100 Expert software. RIN provides a numerical assessment of the integrity of RNA that facilitates the standardization of the quality interpretation; for microarray processing, only RNAs with RIN number >7.5 were further processed to reduce experimental biases due to poor RNA quality.

### Data base construction and probe design

The initial set of ESTs was filtered to remove low quality sequences. The remaining ESTs were masked for vector and repetitive sequences using RepeatMasker software and fish repetitive element database. Expressed sequences were obtained from 17 normalized cDNA libraries, each representing a different tissue (liver, ovary, testis, bone/cartilage, brain/pituitary, heart/vessels, adipose, head/kidney, trunk/kidney, gill, intestine; normal spleen, pathogen-stimulated spleen, muscle; skin, ultimobranchial organ, Stannius corpuscoli). A detailed description of library construction and clone sequencing will be reported elsewhere (Passos et al. in preparation; Ferraresso et al. in preparation). All the ESTs have been submitted to NCBI; the GenBank accession number of the EST showing the highest identity with each cluster is reported in the GEO "Platform data table" (GPL6467). The ESTs together with all publicly available sea bream mRNA sequences were clustered using a strategy based on Blast to identify candidate sequences (cut off e-value set to e-10) to be included in a cluster and Cap3 [45] to perform the assembly and produce the consensus sequences. ESTs were considered to belong to the same cluster if there was an overlap of at least 40 bp and an overlap percent identity of 90%. The clustering pipe-line produced a final set of 19,734 different clusters.

The annotation process was performed using the Blast algorithm. The selection criteria were limited to the best hit with an e-value of at least e-10. The procedure involved two different steps: i) a blastx and a blastn search was performed against a database containing all the predicted and annotated genes in high quality draft genomes of four teleost (*Danio rerio, Gasterosteus aculeatus, Takifugu rubripes *and *Tetraodon nigroviridis*); ii) a blastx of all the genes that did not show any match in the previous step was performed against the amino-acid non redundant database. The gene ontology terms and Pfam ID associations were done only for annotated genes and were performed using UniprotKB as reference database. The clusters with a similarity to an Uniprot entry inherited its gene ontology terms and, when available, its Pfam ID. In order to have an overview of the gene ontology content simplifying the results of the GO annotation, we used the terms of the GOA slim downloaded from the gene ontology web site [46].

The SAPD (SAPD: *Sparus aurata *PaDova) database, based on the BioMart environment, can be queried using different filters based on cluster ID, description, GO, Pfam ID or for a combination of these criteria. It is possible to visualize different attributes choosing among the cluster name, the sequence cluster consensus and GO annotation.

Two non-overlapping probes for each unique transcript were used to construct a high-density sea bream microarray. Probe design was carried out by the Agilent bioinformatic support team that used proprietary prediction algorithms to design oligo-probes, each assigned with a score reflecting the predicted quality of hybridization performance. “Base Composition (BC) content” was used as an indicator of probe quality. BC scores, based on a five grade system (BC1-4, BC poor), were assigned to each probe according to a set of heuristically-derived rules. The two primary aspects of the rules are base composition ratios and “Homeomeric runs”. Base composition ratios represent the percentage of bases (A, T, G, C) in comparison to each other; "Homeomeric runs" are stretches of the probe sequence that contain the same base, reducing probe complexity and increasing the chance of non-specific hybridization, in the appropriate conditions.

### Microarray processing and data analysis

A total of 39,379 oligonucleotide probes were used to construct high-density sea bream microarray based on the Agilent 4 × 44 K design format; the microarrays were synthesized *in situ *using non-contact inkjet technology. Microarray validation was then carried out analyzing the gene expression profile of 19,715 unique transcripts in two early stages of gilthead sea bream development, larvae at one and four days post-hatching. Sample labelling and hybridization were performed according to the Agilent One-Color Microarray-Based Gene Expression Analysis protocol; more details of the followed procedure can be found in Additional file 3.

An Agilent G2565BA DNA microarray scanner was used to scan arrays at 5 μm resolution, *Feature Extraction Software 9.5.1 *was then used to process and analyse array images. The software returns a series of spot quality measures in order to evaluate the goodness and the reliability of spot intensity estimates. Among these measures the *Feature Extraction Software 9.5.1 *flag "glsFound" (set to 1 if the spot has an intensity value significantly different from the local background, 0 otherwise) was used to filter out unreliable probes. From now on those probes with *FeatureExtraction *flag equal to 0 will be noted as "missing". Then, in order to make more robust and unbiased statistical analysis, probes with a high proportion of missing values were removed from the dataset. The proportion of missing value used as threshold in the filtering process was decided according to the experimental set up. Finally, *spike-in *control intensities (*Spike-In *Viral RNAs) were used to identify the best normalization procedure for each dataset. After normalization, *spike *intensities are expected to be uniform across the experiments of a given dataset. On our data cyclic lowess [47] always outperformed quantile normalization.

Pearson correlation coefficients estimated within and among arrays have been used to evaluate array repeatability and precision. Filtering, normalization and correlation analysis were performed using R statistical software [48]. Finally, SAM statistical test was used to identify differentially expressed genes between *S. aurata L*. developmental Stage 1 and 4. A non parametric Spearman rank-correlation test was used to assess correlation between expression values measured respectively with real-time RT-PCR and microarray. The same test was performed separately for each microarray probe. Spearman correlation tests were implemented using SPSS ver. 12.0.

### Gene expression analysis based on real-time RT-PCR

Eleven target genes were selected for real-time RT-PCR analysis. For each selected target gene and for the reference gene (MDH1), a qRT-PCR assay was designed using the Universal Probe Library (UPL) system [49] (RocheDiagnostic, Mannheim, Germany). Gene-specific primers and the most appropriate universal probe were defined for each transcritpt with the ProbeFinder software [50]. To design intron-spanning probes, putative intron-exon boundaries were inferred by comparison with homologs of sea bream genes present in high-quality draft genome sequences from other fish species (*Tetraodon nigroviridis, Danio rerio*, and *Gasterosteus aculeatus*).

One microgram of total RNA for each sample was reverse transcribed to cDNA using Superscript II (Invitrogen™). An aliquot (2.5 μl) of diluted (1:40) cDNA template was amplified in a final volume of 10 μl, containing 5 μl of FastStart TaqMan^® ^Probe Master 2× (Roche Diagnostics), 0.25 μl of each gene-specific primer (10 μM) and 0.1 μl of UPL probe (100 μM). The amplification protocol consisted of an initial step of 2 min at 50°C and 10 min at 95°C, followed by 45 cycles of 10 s at 95°C and 30 s at 60°C. All experiments were carried out in a LightCycler^® ^480 (Roche Diagnostics). To evaluate the efficiency of each assay, standard curves were constructed amplifying two-fold serial dilutions of the same cDNA (sample Sa1A), which was used as calibrator. For each sample, the Cp (Crossing point) was used to determine the relative amount of target gene; each measurement was made in duplicate, and normalized to the reference gene (Malate dehydrogenase 1, MDH1, probe name SAPD02236), which was also measured in duplicate. MDH1 was chosen as reference gene in qRT-PCR assays as it is considered a housekeeping gene, and it did not exhibit any significant change in microarray data between the two developmental stages tested (%CV Probe_1 and Probe_2 of 6.2% and 7.2% respectively). Samples tested in real-time RT-PCR were the same of microarray experiments; one of the biological replicates of Stage 1 (Sa_stage1_A) was used as calibrator, the internal control for each amplification reaction.

## Authors' contributions

LB, TP, and AVMC conceived and designed the project. RR produced the EST sequences. NV conceived and constructed the data base. EN carried out probe design and editing. SF and BC performed microarray experiments. CR edited expression data and carried out all statistical analyses. SF and ANM validated array data with qRT-PCR. LB wrote the manuscript. All listed authors edited the manuscript. All authors read and approved the manuscript.

## Supplementary Material

Additional file 1 **Distribution analysis of hybridization success across 10 microarray experiments**. For each probe, the number of times it was called "present" by Agilent *Feature Extraction 9.5.1 *software was calculated. On the y-axis, the number of positive calls in 10 experiments (0 corresponds to probes that never hybridized, 10 corresponds to probes that always successfully hybridized). On the x-axis is the number of probes falling into each group (0–10). The exact count of probes and the corresponding percentage are also reported for each group. Click here for file

Additional file 2 **List of significant probes identified by SAM analysis**. Up-regulated and down-regulated genes in Stage 4 compared to Stage 1. Click here for file

Additional file 3 **RNA amplification, labelling and array hybridization**. Details of the followed procedure for sample labelling and hybridization. Click here for file
