# Supplementary figures and images for: Development and validation of a gene expression oligo microarray for the gilthead sea bream (Sparus aurata)
Source: BMC Genomics. 2008 Dec 3;9:580. doi: 10.1186/1471-2164-9-580 (PMC2648989; doi:10.1186/1471-2164-9-580)

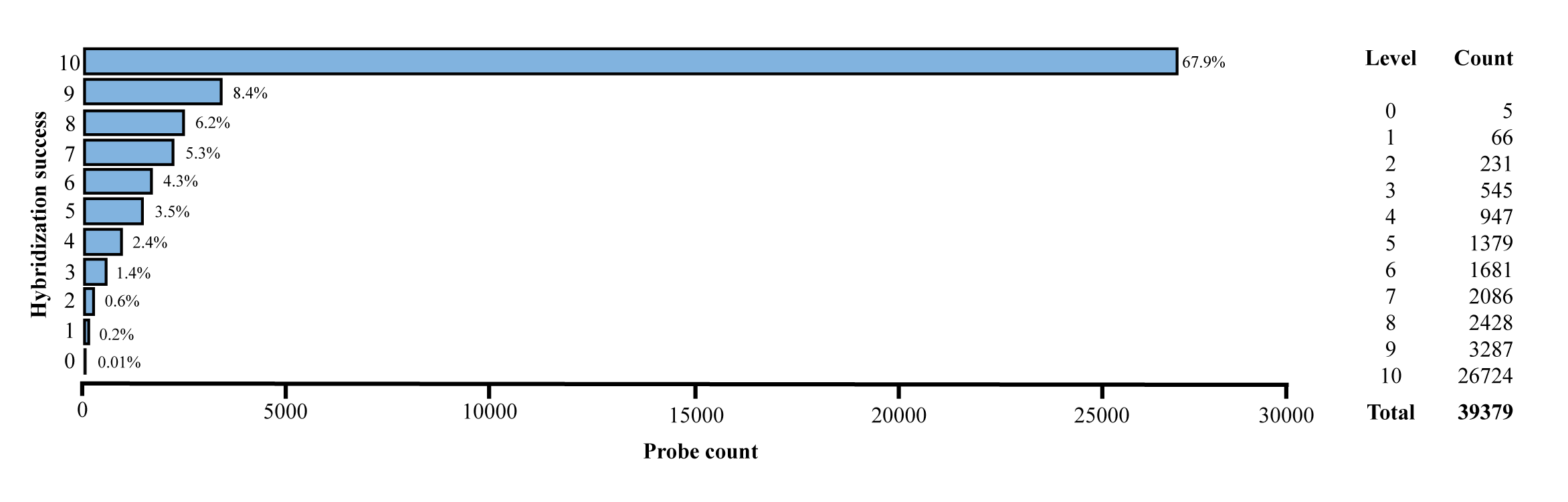

Supplement: Additional file 1 — Distribution analysis of hybridization success across 10 microarray experiments. For each probe, the number of times it was called "present" by Agilent Feature Extraction 9.5.1 software was calculated. On the y-axis, the number of positive calls in 10 experiments (0 corresponds to probes that never hybridized, 10 corresponds to probes that always successfully hybridized). On the x-axis is the number of probes falling into each group (0–10). The exact count of probes and the corresponding percentage are also reported for each group. [file 1471-2164-9-580-S1.tiff]
