# Supplementary material for: Development and validation of a gene expression oligo microarray for the gilthead sea bream (Sparus aurata)
Source: BMC Genomics. 2008 Dec 3;9:580. doi: 10.1186/1471-2164-9-580 (PMC2648989; doi:10.1186/1471-2164-9-580)
Supplement: Additional file 3 — RNA amplification, labelling and array hybridization. Details of the followed procedure for sample labelling and hybridization. [file 1471-2164-9-580-S3.doc]

**RNA amplification, labelling an array hybridization**

Sample labelling and hybridization were performed according to the Agilent One-Color Microarray-Based Gene Expression Analysis protocol.

For each sample, 500 ng of total RNA were linearly amplified and labelled with Cy3-dCTP following the Agilent One-Color Microarray-Based Gene Expression Analysis protocol. A mixture of 10 different viral poly-adenilated RNAs (Agilent Spike-In Mix) was added to each RNA sample before amplification and labelling, to monitor the microarray analysis workflow. Labelled cRNA was purified with the Qiagen RNAeasy Mini Kit, and sample concentration and specific activity (pmol Cy3/µg cRNA) were measured in a NanoDrop® ND-1000 spectrophotometer. A total of 1650 ng of labelled cRNA was prepared for fragmentation adding 11 μl 10X Blocking Agent and 2.2 μl of 25X Fragmentation Buffer, heated at 60°C for 30 min, and finally diluted by addition with 55 μl 2X GE Hybridization buffer. A volume of 100 µl of hybridization solution was then dispensed in the gasket slide and assembled to the microarray slide (each slide containing four arrays). Slides were incubated for 17 h at 65°C in a Agilent Hybridization Oven, subsequently removed from the hybridization chamber, quickly submerged in GE Wash Buffer 1 to disassembly the slides and then washed in GE Wash Buffer 1 for approximately 1 minute followed by one additional wash in pre-warmed (37°C) GE Wash Buffer 2.

After washing the arrays were scanned on a Agilent G2565BA DNA microarray scanner, at a resolution of 5 µm, modifying default settings to scan the same slide twice at two different sensitivity levels (XDR Hi 100% and XDR Lo 10%). The two linked images generated were analyzed together and data were extracted and background subtracted using the standard procedures contained in the Agilent *Feature Extraction Software 9.5.1*.
